# Characterization of bovine-teeth nanoparticles and its effect on BMP-2 expression in periodontitis rat model

**DOI:** 10.1590/1807-3107bor-2026.vol40.018

**Published:** 2026-03-30

**Authors:** Desi Sandra SARI, Shierin Velly FIOLITA, Millenieo MARTIN, Syafika Nuring FADIYAH, Herniyati, Fourier Dzar Eljabbar LATIEF, Peni PUJIASTUTI, Yuliana Mahdiyah Da’at ARINA, Ernie MADURATNA, Haslinda RAMLI

**Affiliations:** (a) Universitas Jember, Faculty of Dentistry, Department of Periodontics, Jember, Indonesia.; (b) Universitas Jember, Faculty of Dentistry, Undergraduated Student, Jember, Indonesia.; (c) Universitas Hasanuddin, Faculty of Dentistry, Department of Oral and Maxillofacial Surgery, Makassar, Indonesia.; (d) Universitas Jember, Faculty of Dentistry, Department of Orthodontics, Jember, Indonesia; (e) Institut Teknologi Bandung, Faculty of Mathematics and Sciences, Physics of Earth and Complex Systems, West Java, Indonesia.; (f) Universitas Airlangga, Faculty of Dental Medicine, Department of Periodontics, Surabaya, Indonesia.; (g) Universiti Sains Islam Malaysia, Faculty of Dentistry, Department of Periodontics, Kuala Lumpur, Malaysia.

**Keywords:** Bone Transplantation, Bone Regeneration

## Abstract

Bovine teeth-derived nanoparticles show strong potential in regenerating alveolar bone tissue damaged by periodontitis. They possess osteoinductive and osteoconductive properties that stimulate cell differentiation, proliferation, and extracellular matrix formation. This study characterized nanoparticles from bovine teeth and evaluated their potential for alveolar bone regeneration via particle size analysis, transmission electron microscopy (TEM), X-ray diffraction (XRD), X-ray fluorescence (XRF), Brunauer–Emmett–Teller (BET) analysis, micro-computed tomography, and Fourier-transform infrared spectroscopy (FTIR). Twenty Wistar rats were divided into four groups: one control and three treatment groups, which were observed on days 7, 14, and 28. The rats were injected with lipopolysaccharides from *Porphyromonas gingivalis* to create a model of periodontitis model. Bovine teeth nanoparticles were applied to the periodontal pockets and covered with a periodontal dressing. The particle sizes ranged from 3.67 to 36.75 nm. TEM images revealed that the particles were spherical, while XRD analysis showed that the components consisted of 63% hydroxyapatite and 37% whitlockite. XRF analysis showed that the mineral content was highest in calcium (81.96%) and phosphorus (15.90%). BET analysis indicated that the characteristics of the nanoparticle material were similar to those of commercial bovine bone material. FTIR analysis revealed the presence of hydroxyapatite groups, including −OH, H_2_O, and P-4. BMP-2 expression levels were notably higher in the repaired bone on day 28 than on days 7 and 14. In conclusion, bovine teeth-derived nanoparticles demonstrate significant potential as biomaterials for alveolar bone regeneration in periodontitis therapy.

## Introduction

Nanomaterials offer promising approaches for tissue and bone repair and regeneration. Over the past few decades, research has focused on the use of nanoparticles for biological applications, including the treatment of periodontal diseases, such as periodontitis. Periodontitis is an inflammatory condition affecting the tissues supporting teeth, leading to progressive resorption of alveolar bone.^1,2^ If untreated, this can result in tooth loss.^3^ Nanoparticles derived from bovine teeth represent a potential regenerative treatment.^4^ Bovine teeth possess osteoinductive properties that stimulate the differentiation of cells into mature osteoblasts, and osteoconductive properties that facilitate cell adhesion, proliferation, and extracellular matrix formation.^5,6^

Bovine tooth scaffolds are effective materials for regenerating bone tissue. Sari et al.^2^ reported that combining stem cells with bovine tooth scaffolds enhances osteogenic differentiation,^2^ evidenced by increased proliferation and differentiation of mesenchymal cells into new bone.^7^ Micro-computed tomography (micro-CT) studies have shown that bovine tooth scaffold composites can promote alveolar bone growth in a periodontitis model of Wistar rats.^2,3^

Sari et al.^7^ also noted that the development of bovine tooth scaffolds is still at the micro-sized level. In vitro studies have shown that hydroxyapatite nanoparticles, particularly those measuring 20 and 80 nm, are more effective in promoting osteoblast proliferation from mesenchymal cells than hydroxyapatite particles measuring 12 μm.^7^ Nanoparticles outperform microparticles owing to their larger surface area, which enhances cell adhesion, proliferation, and differentiation, ultimately improving tissue regeneration.^8^ Moreover, they are easier to apply to periodontal gaps and are more readily absorbed than microparticles.^9^

Nanoparticles with a nanoporous structure provide a greater surface area, which enhances the adsorption efficiency of proteins and growth factors. The development of these nanoparticles offers advantages, including improved adhesion, proliferation, and differentiation properties compared to traditional microparticle-based scaffolds.^10^ They can also promote osseointegration, osteoconduction, and osteoinduction.^11^

In this study, we characterized nanoparticles derived from bovine teeth through various tests, including particle size analysis (PSA), transmission electron microscopy (TEM), X-ray diffraction (XRD), X-ray fluorescence (XRF), Brunauer–Emmett–Teller (BET) analysis, Fourier-transform infrared spectroscopy (FTIR), and assessment of bone morphogenic protein-2 (BMP-2) expression in periodontitis models.

## Methods

The study was approved by the Health Research Ethics Committee of the Faculty of Dentistry at Jember University (approval no. 2145/UN25.8/KEPK/DL/2023). The primary material used was a 1 g bovine tooth scaffold, measuring 355–710 µm, obtained from a preliminary research collection by Sari et al..^2^ The scaffold was derived from the dentin and cementum of crushed cow teeth, processed using a bone mill. The powder was demineralized with 1% HCl for 1 day and subsequently neutralized. Finally, the powder was sterilized and stored using a freeze-drying method.

### Preparation of bovine teeth nanoparticle scaffolds

A 1 g sample of the bovine teeth scaffold, measuring 355–710 µm, was placed in a ceramic milling chamber with ceramic balls of various sizes (8, 10, 15, and 30 mm). The sample was milled for 32 h or until the desired nanoparticle size was achieved. The ground material was separated and sieved using a 200-mesh screen.

### PSA

The powder was sonicated in distilled water to prepare a 10 ppm solution and ensure homogeneity. After achieving a uniform solution, the sample was analyzed using a particle size analyzer (Biobase BK-802N) and the corresponding software.^12^

### TEM assay

The powdered sample was dissolved in distilled water and sonicated for 10 min to achieve homogeneity. A 10 μL sample solution was placed on a grid and allowed to sit for 1 min, after which the excess liquid was removed with a micropipette. Next, 10 μL of uranyl acetate was added, and the excess liquid was removed with a micropipette. The grid was dried for 30 min before analyzing the samples via TEM (Jeol JEM-1400); the imaging results were examined using ImageJ.^13^

### XRD assay

The characteristics of hydroxyapatite derived from bovine teeth were evaluated using XRD equipment, operated at 40 kV and 30 mA. The X-ray radiation source for the machine was copper (Cu), which had an electromagnetic wavelength of 1.54060 Å. Testing commenced with the preparation of nanoparticle samples. The sample was placed in a holder and positioned on the X-ray diffractometer, ready for irradiation and analysis.^14^

### XRF assay

Characterizing the chemical element content using XRF begins with specimen preparation. The same samples were used as in the previous experiments. First, the specimens were smoothed using sandpaper with a grit ranging 200–1,500 until the metal surface was corrosion-free. The specimens were cleaned and analyzed using a Niton XL2 GOLDD testing device, which utilizes XRF; this equipment identifies chemical element variations and determines the percentage of each element in the sample, focusing on the metal surface during analysis. X-rays were emitted, displaying data on a program monitor screen and providing a detailed analysis of the sample composition.^15^

### BET assay

The BET analysis was conducted to determine the surface area, pore volume, and pore size of the sample. The sample was placed in a tube, covered with a heating mantle, and connected to a degassing unit. Degassing eliminated absorbed gases from the solid surface by heating it under vacuum conditions. The analysis began by filling a cooling container with a cryogenic gas, such as nitrogen or carbon dioxide, as the adsorbent.^16^

### FTIR assay

The FTIR test was initiated by injecting or dripping a liquid sample into an infrared cell equipped with a NaCl or potassium bromine crystal window. Subsequently, the prepared sample was placed in the sample area of the interferometer. Measurements were taken until the spectra were displayed on a computer screen.^14^

### Rat model of periodontitis

Twenty male Wistar rats (*Rattus norvegicus*) were divided into four groups (n = 5 per group) according to the formula used by Daniel:^17^


$$n\geq \frac{z^{2}\cdot \sigma^{2}}{d^{2}}$$

*n* = number of samples

*z* = *z* value at a certain error *α*, if *α* = 0.05, then *z* = 1.96

*σ* = standard deviation

*d* = tolerable error

assuming that *σ* ^2^ = *d* ^2^


$$n\geq \frac{z^{2}\cdot \sigma^{2}}{d^{2}}$$

*n* ≥ *z* ^2^

*n* ≥ (1,96)^2^

*n* ≥ 3,84

Corrections are made based on N=n/(1−f)=3,84/0⋅9=4,2 , which is approximately five rats for each group to account for the anticipated deaths in the experimental sample.^18^

The inclusion criteria were as follows: rats weighing between 200 and 250 g, aged 12–14 weeks, and in good health. Rats with physical disabilities, showing signs of disease, and/or with aggressive behavior were excluded from the study.

Before inducing periodontitis with *Porphyromonas gingivalis (P. gingivalis)* lipopolysaccharide (LPS), the experimental animals were anesthetized with a 1:1 mixture of ketamine and xylazine at doses of 40–75 mg/kg and 5–10 mg/kg, respectively, administered intramuscularly into the right posterior thigh. This combination of anesthetics resulted in an effect lasting approximately 20–30 min. The periodontitis model was established by injecting *P. gingivalis* LPS into the interproximal gingiva between the right mandibular first and second molars. Each experimental animal received 10 µL of *P. gingivalis* LPS at a concentration of 0.5 mg/mL, delivered using a 1-cc/mL tuberculin syringe (Terumo) with a 30-G needle (BD). This injection was performed three times a week for 6 weeks.^19,20^ A topical analgesic (3 % lidocaine gel) was applied to the gingiva after every *P. gingivalis* LPS injection to alleviate the pain associated with the injection.

### Nanoparticle administration in a rat model of periodontitis

This study employed the simple random sampling method, in which each sample was assigned a number and selected using a lottery system. As the simplest and most widely used method for selecting samples, this technique ensures that each sample has an equal chance of being selected, allowing researchers to conduct random selections without bias.^21^ The animals were divided into four groups (n = 5 each) comprising one control group (Group I) and three experimental groups (Groups II–IV, which received bovine teeth nanoparticles for 7, 14, and 28 days, respectively). Bovine teeth nanoparticles with a volume of 0.5 mg were placed in the periodontal pocket between the left mandibular first and second molars, and the wound was closed using a periodontal pack (Resopack).^22^

### Decapitation and retrieval of the research specimens

The animals were humanely euthanized on days 7, 14, and 28 by exposure to ether vapor (5 mL applied to cotton in a sealed chamber for 2–3 min per mouse). They were placed in the chamber for approximately 5 min in accordance with the institutional animal care guidelines. After euthanization, the right mandibular bone was immersed in a 10% buffered formalin fixing solution for at least 8 h before decalcification. The rat jaw samples were sent for micro-CT analysis to assess bone growth resulting from the treatment. The specimens were then immersed in ethylenediaminetetraacetic acid (EDTA) solution and observed to ensure that no air bubbles were present. After decalcification, the specimens underwent immunohistochemical examination, and the animals were finally incinerated.^23^

### Expression of BMP-2

Immunohistochemical staining was conducted to examine the expression of BMP-2. The samples were deparaffinized and treated with a 3% hydrogen peroxide solution for 30 min to eliminate endogenous peroxidase activity. Subsequently, they were washed with water, rinsed with distilled water, and treated with phosphate-buffered saline (PBS) for 2 min each. After incubation in a 0.025% trypsin solution in PBS (pH 7.4), the samples were exposed to the mouse antirat BMP-2 monoclonal antibody for 30 min. Next, they were incubated with streptavidin-horseradish peroxidase (HRP) label, followed by the addition of a chromogen substrate (DAB solution). Finally, the samples were mounted in Mayer’s hematoxylin and observed under a light microscope.^24,25^

### Micro-CT assay

The samples were scanned using a high-resolution micro-CT scanner (Bruker Micro-CT SkyScan 1173; Kontich, Belgium), which operates with an X-ray source energy of 40–130 kV. It produces three-dimensional (3D) images of the microstructures by measuring the X-ray attenuation. A minimum scan rotation of 180° is required to create a 3D tomography image; however, the SkyScan 1173 utilizes a rotation of at least 240°, with the rotation step adjustable according to the selected image resolution. The scanner offers three quality options for raw projection images: high-quality (spatial resolution of 5–35 µm), medium-quality (10–70 µm), and standard-quality modes (20–140 µm).

The scanning parameters included a 60-kV energy setting, 106 µA current, 250 ms exposure time, and a 1.0 mm aluminum filter. Scanning was performed using a rotation step of 0.2° and 1 × 1 camera binning, resulting in a raw projection image resolution of 5.70 µm/pixel. After scanning, the NRecon v.1.7.3.1 software (BrukerMicro-CT) was used to generate 3D grayscale images through reconstruction. This process employs the Feldkamp back-projection algorithm in the GPU-based GPUReconServer v.1.7.3.1 (Bruker Micro-CT).^26^

### Data analysis

The research data were analyzed using SPSS version 24. The non-parametric Kruskal–Wallis test was conducted, followed by the Mann–Whitney U test to determine significant differences among groups, with a significance level of p < 0.05.

## Results

This research is divided into two parts: characteristics and in vivo testing.

### Particle size distribution

**Figure 1:**
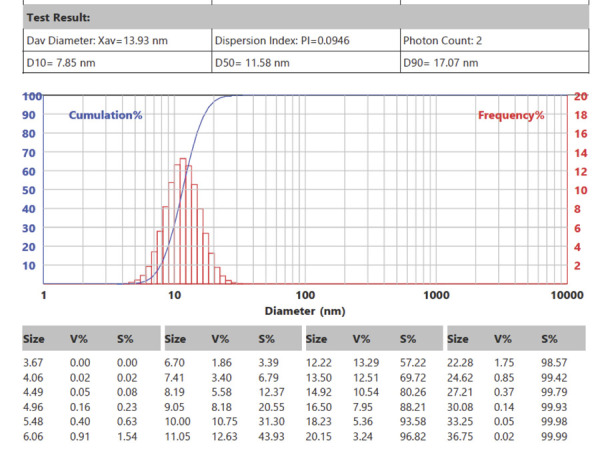
The particle size analysis test results indicated that the particle size distribution of bovine teeth scaffolds categorized as nanoparticles had an average size of 13.93 nm.

The particle size distribution of the bovine teeth nanoparticles is illustrated by a histogram featuring the cumulative and frequency distribution curves. The frequency distribution curve indicated the number of particles within a specific diameter range. The frequency percentage points in the red histogram, referred to as V% in the Table, were connected to form a bell-shaped curve representing the frequency distribution. The cumulative distribution curve provided information about the cumulative percentage of particles or the total number of particles at a specific size. As shown in Figure 1, the cumulative percentage points in the blue histogram, identified in the Table as S%, were connected to form a cumulative particle size distribution curve.

The frequency distribution curve of the particle sizes may be either symmetrical or asymmetrical; therefore, the following measurements were obtained: median = 11.58 nm, mode = 12.22 nm, and mean = 13.93 nm. A difference was observed among the three values, with the values showing the relationship mean > mode > median. Consequently, the particle size distribution of bovine tooth nanoparticles was deemed to be asymmetrically distributed and skewed to the left, indicating that most particles were smaller in diameter than the average value.

### Structure and morphology of bovine teeth nanoparticles in the TEM assay

**Figure 2:**
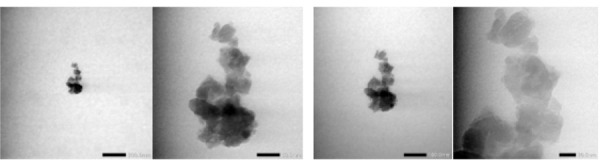
Transmission electron microscopy image of bovine teeth showing relatively round nanoparticles with uneven edges and visible pores (20–200 nm magnification)

The TEM images of the prepared bovine teeth nanoparticles confirmed their spherical shape, with sizes ranging approximately 20–200 nm (Figure 2).

### Component of bovine teeth nanoparticle by XRD assay

**Figure 3:**
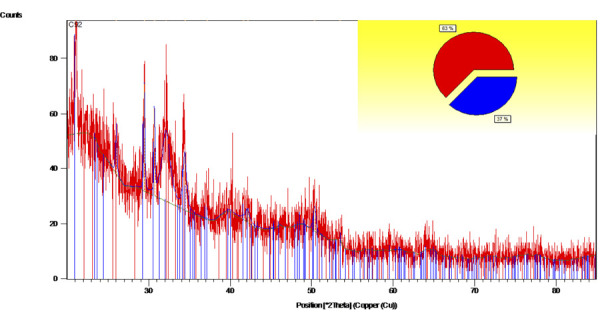
X-ray diffraction spectra of the bovine teeth nanoparticles

The XRD pattern analysis of the bovine teeth nanoparticles revealed that the material was highly crystalline, mainly consisting of hydroxyapatite crystals (63%) along with 37% whitlockite (Table 1). The hydroxyapatite compounds were identified at diffraction angles of 2θ: 21.08°, 21.13°, 23.63°, 25.98°, 32.17°, 42.18°, 63.11°, and 63.93°, with the highest intensity peak being 21.08°. This finding was associated with the absence of total constructive and destructive X-ray interferences in a limited-sized lattice. In addition, inhomogeneous lattice strain and structural defects contributed to the broadening of the diffraction pattern. The high concentration of hydroxyapatite may have enhanced its potential as a bone regeneration material (Figure 3).

**Table 1:** XRD Results of bovine teeth nanoparticle scaffold components.

No	Compound name	Percent (%)	Chemical formula
1.	Hydroxyapatite	63	Ca10(PO4)6OH
2.	Whitlockite	37	C5Ca10H3MgNa0.2O26P5

### Mineral content of bovine teeth nanoparticles by XRF assay

In the bovine teeth nanoparticles, the calcium (Ca) content was highest (81.96 %), followed by phosphorous (P; 15.9 %), nickel (Ni; 0.972 %), strontium (Sr; 0.32 %), barium (Ba; 0.3 %), iron (Fe; 0.17 %), copper (Cu; 0.10 %), cerium (Ce; 0.08 %), rhenium (Re; 0.06 %), and zinc (Zn; 0.04 %) (Table 2). A test was conducted to measure the release of the most abundant minerals, specifically Ca and P, using atomic absorption spectroscopy. The results indicated the following levels of Ca over time: on days 7, 14, and 28, the concentrations were 230, 60, and 73 ppm, respectively. The levels of phosphate released were 749, 609, and 588 ppm on days 7, 14, and 28, respectively (Table 3).

**Table 2:** XRF results of the mineral content of nanoparticles (%).

Compound	P	Ca	Ti	Fe	Ni	Cu	Zn	Sr	Ba	Ce	Re
Conc	15.90	81.96	0.05	0.17	0.97	0.10	0.04	0.32	0.30	0.08	0.06

**Table 3:** Results of Ca and P release in nanoparticle samples for various days.

No	Scaffold samples	Calcium (Ca) release results (ppm)	Phosphate (P) release results (ppm)
1	Nanoparticles day 7	230	749
2	Nanoparticles day 14	60	609
3	Nanoparticles day 28	73	588

### Adsorption characteristics based on the Brunauer–Emmett–Teller assay

**Figure 4:**
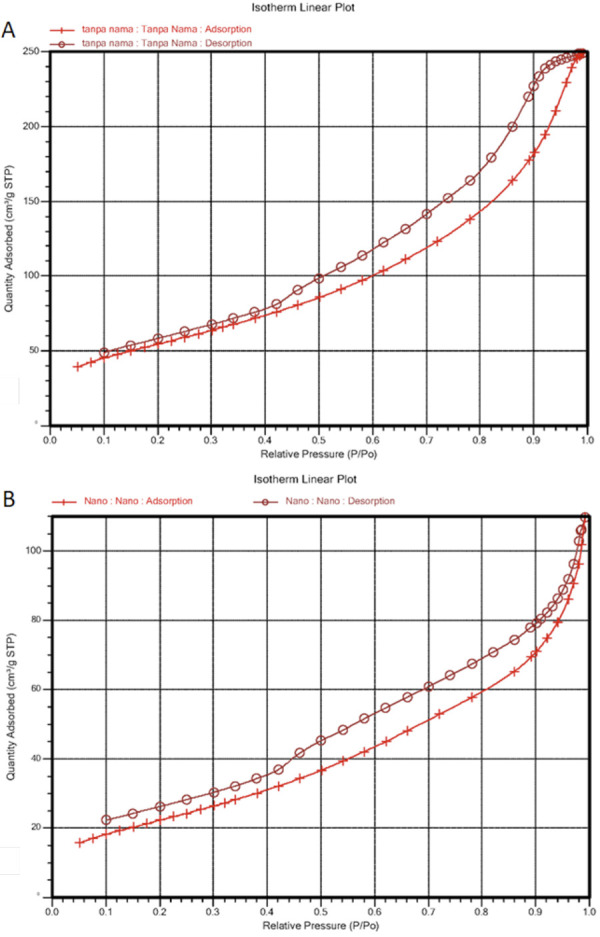
Brunauer–Emmett–Teller assay. A. Bovine bone scaffold (control) B. Bovine teeth nanoparticles

Curves (A) and (B) represent the adsorption characteristics of bovine bone and bovine teeth nanoparticles, respectively. According to the International Union of Pure and Applied Chemistry classification, both curves were classified as Type IV, indicating that they exhibited an adsorption process with mesoporous pores, specifically within a diameter range of 2–50 nm. The average pore diameters of both materials were similar, indicating that they shared the same pore size (Figure 4 and Table 4).

**Table 4:** Bovine teeth nanoparticle BET test results.

Samples	Surface area (m^2^/g)	Pore volume (cm^3^/g)	Adsorption average pore diameter (nm)	Desorption average pore diameter (nm)
Bovine bone fabrication	200,242	0,3846	7,6838	7,0146
Bovine teeth nanoparticles	83,7372	0,167	7,9813	5,8488

### FTIR spectrum

**Figure 5:**
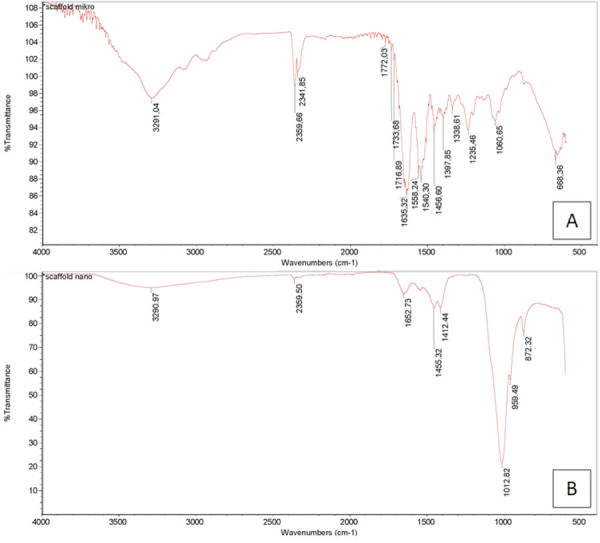
Fourier transformation Infrared spectroscopy image of the bovine bone scaffold (A) and bovine teeth nanoparticles (B)

The FTIR spectrum displayed multiple wavenumbers (Figure 5). The wavenumber at 3,572 cm^–1^ indicated the presence of the O–H group, whereas that at 3,290.97 cm^–1^ corresponded to the H_2_O group, suggesting the presence of an aromatic ring. Additionally, the wavenumber at 1,652.73 cm^–1^ was associated with the P–O group, and that at 959.49 cm^–1^ supported these findings. The results revealed the presence of −OH, H_2_O, and P-O groups, which indicated the presence of hydroxyapatite (Figure 5, Table 5). Reportedly, bovine teeth nanoparticles exhibit hydroxyapatite properties similar to those of hydroxyapatite derived from bovine bone (control).

**Table 5:** Bovine teeth nanoparticle chemical bond.

Chemical bond	Groups	HAP Wavelength (cm^-1^)		Reference (cm^-1^)
		Bovine bone (control)	Bovine teeth nanoparticles	
-OH	OH-structural	3271,7	3,290.97	3,572
H2O	Bending of H2O	1,646	1,652.73	1,644
P-O	PO4 bending	1,014.21	959.49	1,036

### Micro-CT

**Figure 6:**
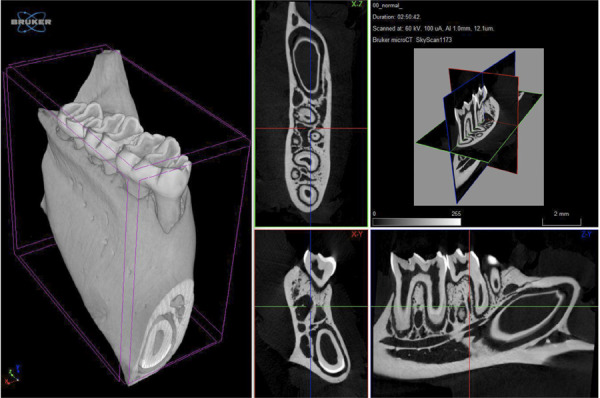
Micro-computed tomography imaging results of nanoparticle administration in a rat model of periodontitis. (A). Control, (B). Group II (received nanoparticles on day 7 [P(7)]). (C) Group III (received nanoparticles on day 14 [P(14)]). (D) Group III (received nanoparticles on day 28 [P(28)])

The micro-CT images presented in Figure 6 indicate that standard samples were used as references to evaluate samples treated with bacteria and subsequently the scaffolds. A defect was observed in samples treated with the scaffold, and the treatment resulted in new tissue growth around this defect. The newly formed tissue was recognized as fibrous. Observations at H7, H14, and H28 revealed that the fibrous tissue exhibited varying thicknesses and growth patterns. By H28, the fibrous tissue became less distinct, whereas the cavities within the trabecular tissue became denser, suggesting that tissue growth can generally be quantified using the trabecular separation parameter (Figure 6).

### Expression of BMP-2

**Figure 7:**
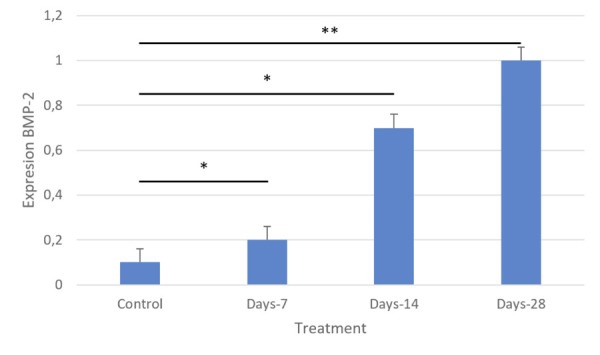
BMP-2 expression was enhanced on day 28 [P(28)] compared to that on days 7 [P(7)] and 14 [P(14)] (*p < 0.05).

**Figure 8:**
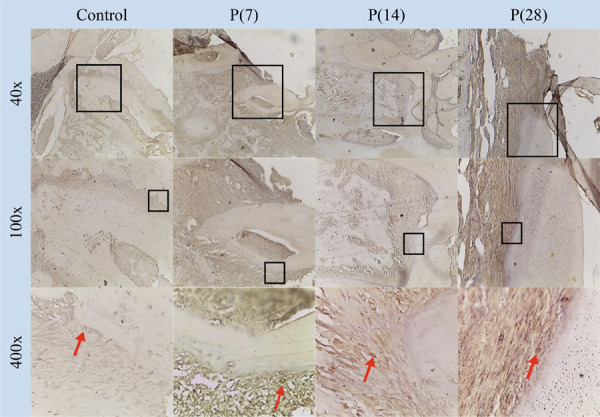
IHC image showing BMP expression in the control and treatment groups on days 7 [P(7)], 14 [P(14)], and 28 [P(28)] at 40×, 100×, and 400× magnification. Red arrows indicate BMP-2 expression.

The results showed a considerable increase in BMP-2 expression in treatment groups II and III compared to the control group. BMP-2 expression was highest in Group IV compared to the other groups (Figure 7). IHC staining revealed BMP-2 expression around the alveolar bone of rats, showing images of osteoblasts that indicate cell proliferation and new bone growth (Figures 6 and 8).

## Discussion

Nanoparticles derived from bovine teeth can enhance regenerative potential in periodontitis therapy by offering several key advantages over synthetic scaffolds. Bovine teeth naturally contain hydroxyapatite and dentin-like structures that closely resemble human bone and tooth tissue, offering a more biomimetic composition. As a biologically derived material, bovine teeth scaffolds demonstrate superior biocompatibility, allowing them to integrate more effectively with host tissues and minimizing the risk of immune rejection. When processed into nanoparticles ranging from 4 to 36 nm, these scaffolds promote enhanced cellular responses, including improved adhesion, proliferation, and differentiation, which collectively accelerate alveolar bone regeneration. Furthermore, their inherent osteoconductive properties create an ideal environment for bone-forming cells, supporting new bone growth within periodontal defects. In addition, the natural composition of these scaffolds contributes to a better modulation of inflammatory responses compared to synthetic alternatives, thereby facilitating a more efficient healing process.

The dual representation in Figure 1—through frequency and cumulative distribution curves—provides a comprehensive understanding of particle dispersion across different size ranges. This variation is particularly notable in biomedical scaffold applications, where a uniform particle size can significantly affect the porosity, surface area, and bioactivity. Smaller particles may enhance cellular uptake, increase surface reactivity, and facilitate controlled release, which is particularly beneficial in drug delivery and regenerative therapies. Conversely, even smaller amounts of larger particles can enhance mechanical reinforcement or create heterogeneous pore formations, which can be beneficial in certain scaffold designs.

The size range can significantly influence the physicochemical properties of the particles, including surface area, surface charge, and contact angle, which can hinder the effectiveness of bone tissue regeneration.^27^ Torres et al. (2022) have shown that hydroxyapatite nanoparticles measuring 20 and 80 nm more effectively enhance mineral deposition than hydroxyapatite particles measuring 12 μm.^28^ In the study by Zhang et al.,^27^ 20-nm hydroxyapatite nanoparticles and 80-nm naHAP—particularly the 20-nm particles—promoted increased osteogenic differentiation of mesenchymal cells. This was attributed to the effect of particle size on key physicochemical characteristics, such as the surface area, contact angle, and surface charge, which can alter the biological effects of the particles. Smaller particle sizes usually lead to larger surface areas, resulting in considerable protein adsorption on their surfaces. Nano-sized particles can bind to proteins on the cell surface in remarkably large quantities.^29^

Nanoparticles are generally formed by the agglomeration of several primary particles—referred to as secondary particles—due to weak surface forces, such as van der Waals or capillary forces, which lead to soft agglomerates, or stronger chemical bonds that result in hard agglomerates. The spherical morphology and nanoscale dimensions observed via TEM suggest improved dispersibility and interaction with cellular membranes, which may enhance osteogenic differentiation.

The release of Ca and P plays a crucial role in the activation of osteoblasts and osteoclasts, facilitating bone regeneration.^30^ Calcium phosphate exhibits osteoconductive and osteoinductive properties that promote the osteogenic differentiation of mesenchymal stem cells, significantly influencing bioactivity and affecting cell adhesion, proliferation, and new bone formation in osteoblasts.^31,32^ Understanding the degradation of calcium phosphate and the release of ions is necessary to demonstrate these bioactive qualities. This process increases the local concentration of calcium and phosphate ions, stimulating the formation of bone minerals on the calcium phosphate surface. In addition, it affects the expression of osteoblastic differentiation markers, including BMP-2.^30^ Calcium phosphate is vital for cell adhesion and tissue formation because it influences the adsorption of extracellular matrix proteins on its surfaces. Its properties also impact bone regeneration by affecting the development of newly formed bone minerals.^33^

FTIR spectra confirmed the presence of functional groups typical of bone apatite, supporting the biomimetic nature of bovine tooth nanoparticles. Human bones are recognized as a biocomposite comprising an inorganic phase embedded within the organic phase of collagen.^34^ Furthermore, the marked upregulation of BMP-2 in the treatment group confirms that bovine teeth nanoparticles enhance osteogenic signaling, consistent with prior findings on hydroxyapatite-based biomaterials. It refers to the activation of the extracellular signal-regulated kinase signaling pathway, which stimulates the expression of osteoblast-specific biomarkers, including BMP-2, that regulate bone growth and development.^35,36^ The finding of increased BMP-2 regulation was also strengthened by the micro-CT results, which showed signs of new bone tissue growth around the defect in the treatment group.

A limitation of this study was that we only conducted qualitative observations using micro-CT. Various other tests should be conducted to quantitatively assess new tissue growth, enabling future research to present the potential of bovine tooth nanoparticles more precisely.

## Conclusion

Bovine teeth nanoparticles exhibit remarkable potential as scaffold materials in bone tissue engineering, particularly for periodontal regeneration, due to their favorable structural, chemical, and biological properties.

## Data Availability

The authors declare that all data generated or analyzed during this study are included in this published article.
